# Clozapine in severe psychotic disorders: Balancing safety with efficacy

**DOI:** 10.1192/j.eurpsy.2021.2053

**Published:** 2021-08-13

**Authors:** R. Almeida Leite, A. Costa, J. Borges, A. Mesquita

**Affiliations:** Psychiatry And Mental Health, Baixo Vouga Hospital Centre, Aveiro, Portugal

**Keywords:** clozapine, side effects, resistant schizophrénia, EFFICACY

## Abstract

**Introduction:**

Clozapine is a member of the dibenazepine class of antipsychotic drugs and has been designated an atypical antipsychotic drug. Clinical studies have shown that clozapine is effective in ameliorating the core symptoms, as well as the negative symptoms, in severe psychotic disorders and is therapeutically effective in treating about 30% of schizophrenic patients who are resistant to standard antipsychotic drugs.

**Objectives:**

The goal is to review pharmacology, efficacy, and clinical use of clozapine, such as its side effects, and the benefit-to-risk ratio of this antipsychotic drug.

**Methods:**

Non-systematic literature review based on scientific databases such as PubMed, using key words such as “clozapine”, “efficacy”, “side effects” and “resistant schizophrenia”.

**Results:**

Clozapine was developed as the first atypical antipsychotic with activity for both the negative and positive symptoms of schizophrenia. The primary indications for clozapine are treatment-resistant psychotic disorder, defined as persistent moderate to severe delusions or hallucinations despite two or more clinical trials with other antipsychotic drugs, and patients who are at high risk for suicide. Concerns over a number of safety considerations are responsible for much of the underutilization of clozapine, such as agranulocytosis, metabolic side effects and myocarditis. These side effects can be detected, prevented, minimized and treated, but there will be a very small number of fatalities.

**Conclusions:**

Awareness of the benefits and risks of clozapine is essential for increasing the use of this lifesaving agent.

**Disclosure:**

No significant relationships.

